# Immunoregulatory function of *Dictyophora echinovolvata* spore polysaccharides in immunocompromised mice induced by cyclophosphamide

**DOI:** 10.1515/biol-2021-0055

**Published:** 2021-06-21

**Authors:** Chenqiang Lin, Hui Zhang, Longjun Chen, Yu Fang, Jichen Chen

**Affiliations:** Fujian Academy of Agricultural Sciences, The Soil and Fertilizer Institute, Fuzhou 350013, People’s Republic of China

**Keywords:** immunomodulatory activity, *Dictyophora echinovolvata*, spore polysaccharides

## Abstract

The purpose of this study was to investigate whether the *Dictyophora echinovolvata* spore polysaccharides (DESP) affect the immunity in immunocompromised mice induced by cyclophosphamide (CTX). The healthy female Kunming mice were randomly divided into six groups, including a normal control (NC) group, a positive control group, a model control (MC) group, and three groups treated with low-, intermediate-, and high-dose polysaccharide, respectively. A series of immunoregulatory properties were determined, including humoral and cellular immunity, immune function, and immune factors of mononuclear macrophages. Compared with NC and MC groups, treatment with DESP significantly increased the spleen index and decreased the thymus index; increased the serum concentrations of immunoglobulin (Ig)A, IgG, IgM, hemolysin, IL-1β, and IL-2; delayed the allergic reaction; and improved the splenic lymphocyte transformation ability; and enhanced the phagocytosis of macrophages and the ability to secrete IL-6, TNF-α, caspase-1, and NO with DESP supplementation. These results indicated that DESP might have a good regulatory effect on CTX-induced immunodeficiency in mice, adjust the body’s immune imbalance, and improve the symptoms of low immunity.

## 1 Introduction

*Dictyophora indusiata* is a stinkhorn fungus that belongs to Eumycota, Basidiomycotina, Basidiomycetes, Phallales, and Phallaceae families [1,2]. It is edible as a delicacy in China, has a long history [3], and is called “queen of the mushrooms” due to its beautiful appearance and delicious taste [4]. This mushroom is also rich in vitamins and micro minerals, has a lot of health benefits to the eyes, acts as a tonic to cardiovascular systems, and shows partially medicinal effects like mental tranquilization and antitumor activity [5,6,7,8]. Polysaccharides are a kind of natural polymer linked by aldose or ketose through glycosidic bonds. As one of the fundamental substances to maintain the normal function of life, polysaccharides are essential biological macromolecules *in vivo* [1,9].

The pileus of *D. indusiata* is covered with olive green mucus, which contains the basidiospore that includes all germplasm of *D. indusiata.* The polysaccharides from the volva of *Dictyophora rubrovolvata* were extracted by hot water, and one of them had an inhibitory effect on the tumor cells of S180 to some extent [6]. As in the previous study, *D. indusiata* polysaccharide could inhibit the immunosuppressive function of cancer-associated fibroblasts [10]. *Dictyophora echinovolvata* Zang is one of the species of *D. indusiata*. At present, it has been the most important commercialized species that occupy most of the market shares of *D. indusiata* in China due to its easy cultivation, high nutritional value, and biological function [11]. The mature fruiting body of *D. indusiata* can be divided into three parts: the pileus covered with spores, the volva, and the edible part, which consists of the stipe and white net-like veil [2]. The edible part of the fruiting body of *D. indusiata* is very delicious and expensive. The volva and pileus that account for 65% of the whole mushroom (by fresh weight) are dumped without use and may cause environmental pollution and resource wastage [12]. The new study has reported that water-extractable polysaccharides by *D. indusiata* play a vital role in the process of antioxidation, hepatic-, and renal protection on obese mice [13]. In addition, previous research also provided the similar evidences associated with the polysaccharide such as *Lonicera japonica* and *Ganoderma lucidum* [14,15,16,17,18]. *L. japonica* polysaccharide was used to analyze the immune regulation function in immunosuppressed mice induced by cyclophosphamide (CTX) in order to better develop this Chinese herbal medicine plant [15]. Other studies have reported that the spores of *G. lucidum* were confirmed to have various biological functions [16,17,18]. These results indicated that the nutritional and biological functions of this abundant waste needed to be thoroughly evaluated to realize the highest possible economical profit [19,20].

However, the spores of *D. indusiata* polysaccharides associated with the immunity regulated function are not clean, especially *D. echinovolvata*. Previous results have shown that the crude polysaccharide fraction of edible *D. echinovolvata* might be a potential function for preventing neurodegenerative diseases in which oxidative stress and apoptosis were involved [21]. In our lab, we isolated and characterized polysaccharides from *D. echinovolvata* spore. The objective of this study was to conduct a qualitative analysis of *D. echinovolvata* spore polysaccharides (DESP) and explore and investigate its immunomodulatory function in CTX-induced immunosuppressed mice models. The ameliorative effects of DESP were estimated by organ index, splenic lymphocyte proliferation, macrophage phagocytosis, and serum cytokines.

## 2 Materials and methods

### 2.1 Polysaccharides and reagents

DESP were prepared in the laboratory. The fruiting body of *D. echinovolvata* was purchased from a local commercial market in Shunchang, Fujian, China. The homogenate of *D. echinovolvata* gelatin was boiled in distilled water for 2 h at a designed temperature. After centrifugation to remove debris fragments, the supernatant was concentrated by rotary evaporation. Protein was removed and collected as the crude polysaccharide fraction of *D. echinovolvata* gelatin. The gelatin was obtained through precipitation with four volumes of 95% ethanol, centrifugation, and freeze-drying.

CTX and levamisole hydrochloride were obtained from Baxter International Co., Ltd. and 20% sheep red blood cell (SRBC) was obtained from Nanjing Senbega Biotechnology Co., Ltd. The fetal bovine serum was obtained from Zhejiang Tianhang Biotechnology Co., Ltd. RPMI-1640 medium is the product of GE Medical Life Science Co., Ltd.; NP-40, knife bean globulin (ConA) is a Sigma product; Hank’s solution, MTT kit, and neutral gum are products of Beijing Solebo Technology Co., Ltd., whereas Penicillin (10,000 U/mL) and streptomycin (10,000 μg/L), 1:1 (v/v) acetone–methanol fixing solution, 4% (v/v) Giemsa-phosphate buffer, 0.1% sodium carbonate solution, heparin, and ink were prepared by us. Methanol, acetone, sodium carbonate, Giemsa dye solution, and heparin were obtained from Beijing Dingguo Changsheng Biotechnology Co., Ltd.

### 2.2 Experimental design and samples

Female, 7-week-old Kunming mice were obtained from SLAC Laboratory Animal Company (Shanghai, China, certificate no.: CXK [Shanghai] 2003-0003). After 1 week of acclimatization to the animal laboratory with drinking and feeding freely, all mice were randomly assigned to six groups, including a normal control (NC) group, a positive control (PC) group, a model control (MC) group, and three groups treated with DESP (low-, intermediate-, and high-dose; 400, 800, and 1,600 mg/kg day, respectively; LP, MP, and HP) for 4 months. The PC group was given levamisole hydrochloric acid with 25 mg/kg day intragastrically. The NC and MC groups were given saline intragastrically. The gavage volume of mice in each group was 0.1 mL (10 g/bw). On the 21st day, except for the NC group, all mice were intraperitoneally injected with 50 mg/kg day CTX for 1 week to develop low immunity. The mice in the NC group were injected with the same amount of saline. The mice were fed under controlled environmental conditions (temperature: 21–23°C; humidity: 40–60%) and a 12 h light/dark cycle.


**Ethical approval:** The research related to animal use has been complied with all the relevant national regulations and institutional policies for the care and use of animals and were approved by the Fujian Provincial Animal Care and Use Committee and the Fujian province Zoological Society.

### 2.3 Organ index

At the end of the experimental period, the mice, fasted without water for 12 h, were weighed and sacrificed after cervical dislocation. The spleen, thymus, and liver samples were collected, and excess fascia and adipose tissue were removed simultaneously. Then, the organs were weighed, and the immune organ index was calculated according to the following formula:

\text{organ}\hspace{.5em}\text{index}\hspace{.5em} \% =\text{organ}\hspace{.5em}\text{weight}\hspace{.5em}(\text{spleen,}\hspace{.5em}\text{thymus,}\hspace{.5em}\text{liver})/\text{body}\hspace{.5em}\text{weight}\times 100 \% \text{.}]

### 2.4 Delayed allergic reaction

After lavage 24 days in mice, they received intraperitoneal injections of 0.2 mL of 5% (v/v) SRBC. The thickness of the metatarsal part of the left posterior foot of mice was measured by a thickness gauge 4 days after immunization with 5% (v/v) SRBC. The average value was obtained by measuring the thickness of the metatarsal part of the left posterior foot of mice three times. Then, 20% of 20 μL (v/v) SRBC was subcutaneously injected at the measurement site. The thickness of the left posterior metatarsal was measured 24 h after injection. The same measurement was made three times, and the average was taken. Mice vola pedis thickening is equal to the average thickness of the second vola pedis minus the average thickness of the first vola pedis.

### 2.5 Splenic lymphocyte transformation experiment

The mice were sacrificed through cervical dislocation, and they soaked in 75% alcohol for 5 min. The spleen was exposed through an abdominal incision and then isolated, collected, and put into a petri dish containing 5 mL Hank’s solution. Subsequently, the spleen was placed on a 200-mesh stainless steel net and placed in a small dish containing the proper amount of aseptic Hank’s solution to make a single-cell suspension. After filtration with a 200-mesh screen, the suspension was washed with Hank’s solution three times. The target cell concentration was achieved by diluting to 1 × 10^6^ cell/mL in RPMI-1640 medium containing 10% fetal bovine serum. The cell suspension 200 μL/well was added to the 96-well cell culture plate. Each splenic cell suspension was divided into experimental group and control group. The ConA solution (final concentration 5 μg/mL) was used in the experimental group. They were cultured in an incubator at 37°C with 5% CO_2_ for 48 h. The 110 μL of DMSO was added into the cell to terminate reaction. The OD value was measured by an enzyme labeling instrument at 570 nm. The proliferation capacity of lymphocyte A is equal to the average OD value of the experimental group minus the average OD value of the control group.

### 2.6 Serum hemolysin and cytokine determination

The concentration of serum hemolysin, immunoglobulin (Ig) A, G, and M, interleukin-1β, and interleukin-2 was determined by ELISA kit (Langton, Shanghai, China) according to the manufacturer’s instruction.

### 2.7 Macrophage phagocytosis

The blood was collected from the inferior pterygoid vein of a chicken with an aseptic blood collection needle for obtaining chicken red blood cells (CRBCs). Also, 2% (v/v) CRBC suspension was prepared with normal saline after centrifuging at 1,000 rpm for 10 min. Mice were intraperitoneally injected with 2% CRBC suspension. After 30 min, the mice were sacrificed by cervical dislocation, and the peritoneal macrophages after intraperitoneal injection of 2 mL of normal saline were collected. Peritoneal macrophages were prepared as described in previous studies [22,23]. After dying in Giemsa-phosphate buffer for 3 min, the number of semi-swallowed red blood cells, total phagocytes, and total macrophages were counted using an optical microscope. The phagocytosis index and phagocytosis rate were calculated based on the formula. The percentage of phagocytosis (%) and tosis index were analyzed by the following formula: \text{the}\hspace{.5em}\text{percentage}\hspace{.5em}\text{of}\hspace{.5em}\text{phagocytosis}=\frac{{N}_{1}}{{N}_{\text{c}}}] and \text{the}\hspace{.5em}\text{tosis}\hspace{.5em}\text{index}=\frac{{N}_{2}}{{N}_{\text{c}}}], where *N*_1_ is the number of macrophages that engulf CRBCs, *N*_2_ is the number of CRBCs consumed, and *N*_c_ is the number of macrophages.

### 2.8 Carbon clearance test

A total of 0.2 mL ink (diluted with saline at 1:4) was injected intravenously into the tail vein of mice. After inoculating ink, blood was sampled and put into a heparin tube from the eyeball of mice at 5 and 18 min, respectively. Then, 20 μL of blood samples was added into 2 mL of 0.1% sodium carbonate solution with a tube for accuracy and analyzed in a spectrophotometer at 600 nm. Finally, the mice were killed by cervical dislocation, and the spleen and liver samples were collected. They were weighed and recorded accurately. The carbon clearance index *α* was calculated according to the following formula:

\begin{array}{c}K=\frac{\mathrm{lg}\hspace{.25em}{\text{OD}}_{1}-\hspace{.25em}\mathrm{lg}\hspace{.25em}{\text{OD}}_{2}}{{t}_{1}-{t}_{2}},\\ \text{Carbon}\hspace{.5em}\text{clearance}\hspace{.5em}\text{index}\hspace{.5em}\alpha =\frac{{W}_{1}}{{W}_{2}}\times \sqrt[3]{K}\text{,}\end{array}]

where *W*_1_ is the body weight and *W*_2_ is the weight of liver and spleen.

### 2.9 Data analysis

The experimental data were analyzed by the least significant difference model of ANOVA with SPSS 20.0 software. Each repetition was used as a statistical unit of the experiment, and the results were expressed as mean ± SEM.

## 3 Results and discussion

### 3.1 Effects on body weight and organ index in immunocompromised mice

**Figure 1:**
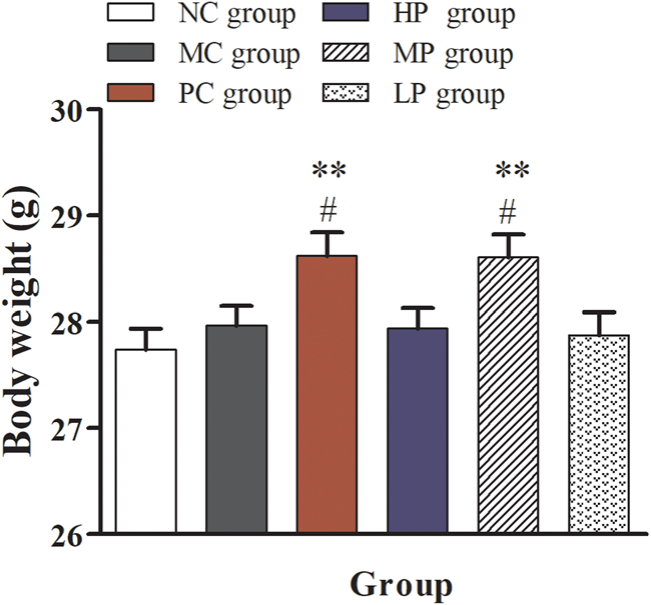
Effect of DESP on the weight of immunosuppressed mice. Compared with the NC group, ^*^ *P* < 0.05 and ^**^ *P* < 0.01; compared with the MC group, ^#^ *P* < 0.05.

As shown in Figure 1, compared with the NC group, the bodyweight of mice was significantly increased (*P* < 0.05) in the PC group and the MP group (*P* < 0.05). The results showed that the treatment of the middle-dose DESP and levamisole hydrochloric acid could achieve the same effect, which further indicated that the treatment of the middle-dose DESP had the effect of promoting immunity similar to that of levamisole hydrochloric acid.

**Figure 2:**
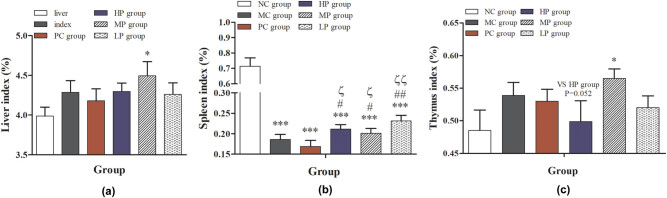
Effect of DESP on organ index in immunocompromised mice: (a) liver index, (b) spleen index, and (c) thymus index. Compared with the NC group, ^*^ *P* < 0.05, ^**^ *P* < 0.01, and ^***^ *P* < 0.001; compared with the MC group, ^#^ *P* < 0.05 and ^##^ *P* < 0.01; compared with the PC group, ^ζ^ *P* < 0.05 and ^ζζ^ *P* < 0.01.

As shown in Figure 2, compared with the NC group, the liver index in the MP group was significantly higher than that of the other groups (*P* < 0.05); the spleen index was significantly decreased in the MC, PC, LP, MP, and HP groups (*P* < 0.001 or *P* < 0.001). These results indicated that the immunocompromised mice model was successfully constructed. Compared with MC and PC groups, the spleen index in the LP, MP, and HP groups was significantly increased (*P* < 0.05 or *P* < 0.01). Meanwhile, the thymus index was also significantly increased in the group treated with the middle-dose DESP (*P* < 0.05). These results suggested that DESP intragastrically could increase the weight of important immune organs like the spleen and thymus gland in mice, thereby affecting the immune function. Although DESP treatment increased the index of the spleen and thymus, the organ atrophy caused by low immunity was irreversible. Thymus and spleen are the main immunization organs. The spleen is the largest peripheral immune organ innervated with sympathetic nerves and controlled by the adrenomedullary system in the body [24]. A bigger immunity index indicates a stronger immune capability [25]. The results of the thymus index and spleen index indicated that DESP could enhance the cell-mediated immunity and stimulate T cell formation [26].

### 3.2 Effect of DESP on cellular immune function in immunocompromised mice

**Figure 3:**
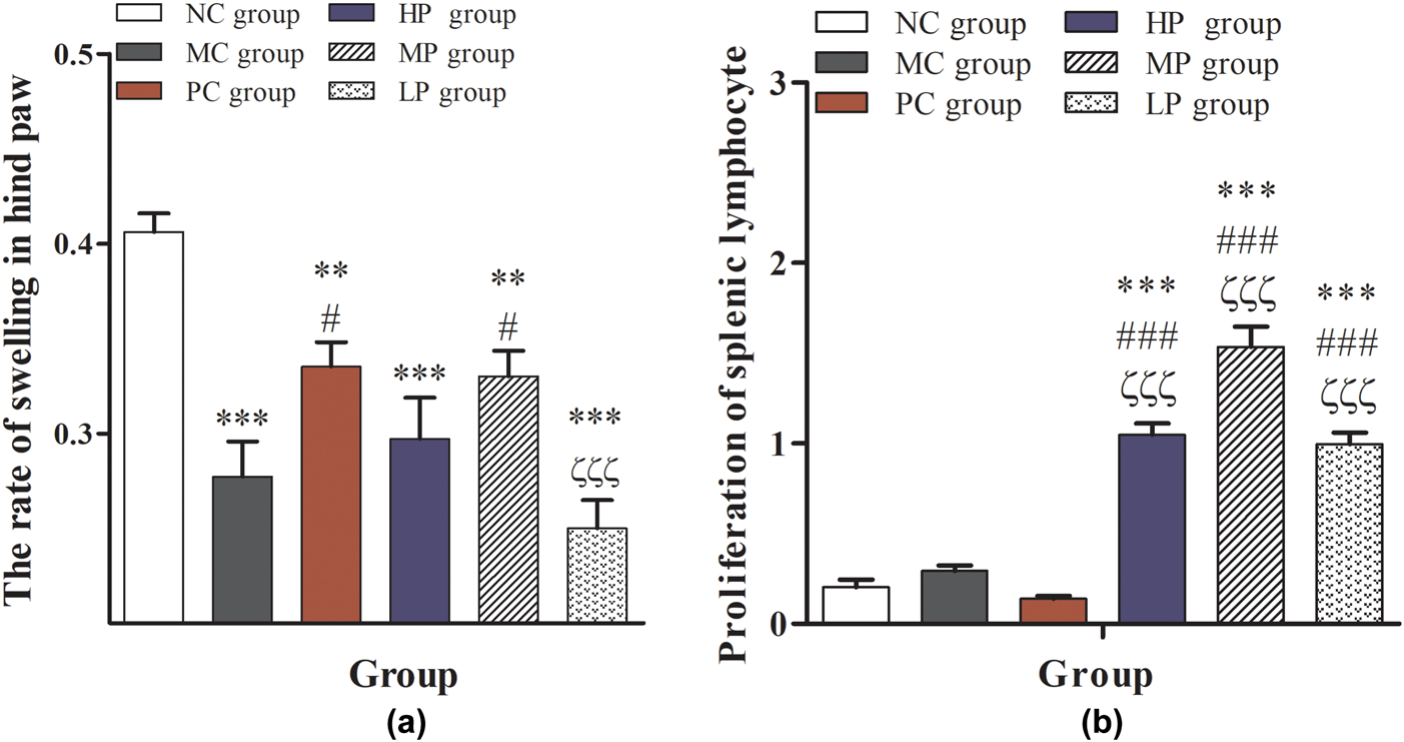
Effects of DESP on delayed allergic reaction and the splenic lymphocyte transformation ability in immunocompromised mice: (a) the rate of swelling in hind paw and (b) proliferation of splenic lymphocyte. Compared with the NC group, ^*^ *P* < 0.05, ^**^ *P* < 0.01, and ^***^ *P* < 0.001; compared with the MC group, ^#^ *P* < 0.05, ^##^ *P* < 0.01, and ^###^ *P* < 0.001; compared with the PC group, ^ζ^ *P* < 0.05, ^ζζ^ *P* < 0.01, and ^ζζζ^ *P* < 0.001.

As shown in Figure 3a, compared with the NC group, the rate of swelling in the hind paw was significantly reduced (*P* < 0.01 or *P* < 0.001). Compared with the MC group, the rate of swelling in the hind paw in the PC group and the MP group was significantly increased (*P* < 0.05), which further indicated that the middle-dose DESP treatment could improve the immunity of immunocompromised mice. Figure 3b shows that the DESP treatment could significantly increase the proliferation and transformation of lymphocytes in mice, especially in the MP group (*P* < 0.001). These results suggest that an intermediate dose of DESP can improve the delayed allergic reaction and the splenic lymphocyte transformation ability and improve the immunity of immunocompromised mice. Previous studies have shown that splenic lymphocyte has an important role during the process of cell-mediated immunity based on the CD4^+^, CD8^+^, and invariant natural killer T (iNKT) cell [27,28,29]. The iNKT cells are a distinct population of innate T lymphocytes selected in the thymus [30,31]. They have an important function of rapidly secreting cytokines and an innate T cell population capable of activating and steering adaptive immune responses [28]. Previous reports have shown that CTX suppressed both humoral and cellular immune responses [32]. The present study showed that treatment with DESP could significantly increase the splenic lymphocyte transformation ability (Figure 3b). This result may suggest that DESP may be capable of improving immunity.

### 3.3 Effects of DESP on the humoral and cellular immune factor

**Figure 4:**
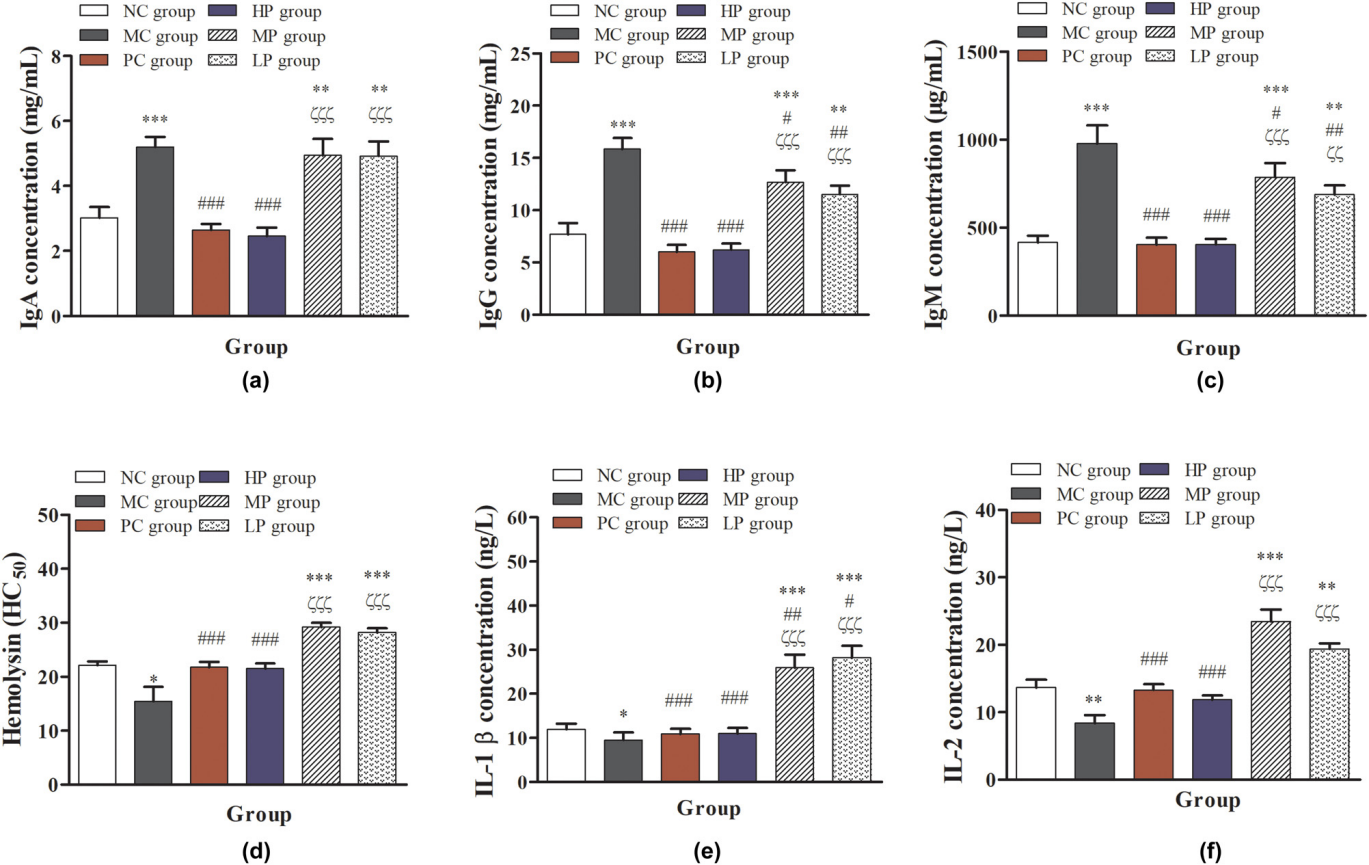
Effects of DESP on the serum concentration of Ig: (a) IgA contents, (b) IgG contents, (c) IgM contents, (d) hemolysin, (e) IL-1β, and (f) IL-2 in immunosuppressed mice. Compared with the NC group, ^*^ *P* < 0.05, ^**^ *P* < 0.01, and ^***^ *P* < 0.001; compared with the MC group, ^#^ *P* < 0.05, ^##^ *P* < 0.01, and ^###^ *P* < 0.001; compared with the PC group, ^ζ^ *P* < 0.05, ^ζζ^ *P* < 0.01, and ^ζζζ^ *P* < 0.001.

As shown in Figure 4, compared with the NC group, the serum concentrations of IgA, IgG, and IgM in the MC, MP, and LP groups were significantly or immensely significantly increased (*P* < 0.05 or *P* < 0.001), but they were significantly decreased (*P* < 0.001) in the PC and HP groups. Compared with the MC group, the serum contents of IgA, IgG, and IgM in PC, HP, MP, and LP groups were significantly decreased (*P* < 0.001). However, the serum contents of IgA in MP and LP show a downward trend. Compared with the PC group, the serum contents of IgA, IgG, and IgM in MP and LP groups were significantly increased (*P* < 0.05 or *P* < 0.01). Based on the present results, they suggested that the intermediate- and low-dose of DESP can stimulate the body to produce higher concentrations of Ig, especially the intermediate-dose of DESP treatment. Immune deficiencies appear to exhibit antibody production deficiencies [33]. In some cases, Ig replacement treatment is the most effective treatment in primary immunodeficiency diseases [34,35,36]. In the present study, DESP treatment significantly increased IgA, IgG, and IgM concentration, and we speculate that DESP can effectively alleviate the damage of immune deficiency to protect immunocompromised mice.

CTX treatment reduced the concentration of hemolysin, IL-1β, and IL-2 significantly in the MC group, compared with the NC group, as shown in Figure 4d–f (*P* < 0.05 or *P* < 0.01). Compared with NC and MC groups, the contents of hemolysin, IL-1β, and IL-2 were significantly increased (*P* < 0.01 or *P* < 0.001) in the MP and LP groups. Compared with the PC group, the concentrations of hemolysin, IL-1β, and IL-2 were significantly increased (*P* < 0.001) in the MP and LP groups. Hemolysin as an index was used to evaluate the body’s immune function by the degree of aggregation of SRBC. IL-1β is produced by activated macrophages, belonging to a cytokine type that can stimulate the proliferation and differentiation of immune response cells and improve its function [8,27]. IL-2 can affect the immune system by affecting T cells and promoting the proliferation of activated B cells, participating in antibody response, detecting tumor and hematopoiesis [29,37]. In addition, the previous results have shown that the *D. echinovolvata* polysaccharide could inhibit the mitochondria-dependent apoptotic pathway to protect against H_2_O_2_-induced neurotoxicity in PC12 cells [21]. Apoptosis and oxidative stress are important features of chronic diseases, including neurodegenerative disorders and immunocompromised individuals [38]. The results showed that the hemolysin in serum of immunocompromised mice induced by CTX was significantly decreased after SRBC injection, indicating that the activity of B cells in mice was inhibited. After intermediate- and low-dose DESP treatment, the hemolysin was significantly increased, which effectively increased the proliferation and antibody production of B cells in mice. In addition, intermediate- and low-dose DESP treatment can significantly increase the contents of IL-1β and IL-2 in immunocompromised mice, suggesting that DESP treatment may promote the activity of macrophages and proliferation of B cells *in vivo*, improve the immune defense ability, and prevent various chronic diseases.

**Figure 5:**
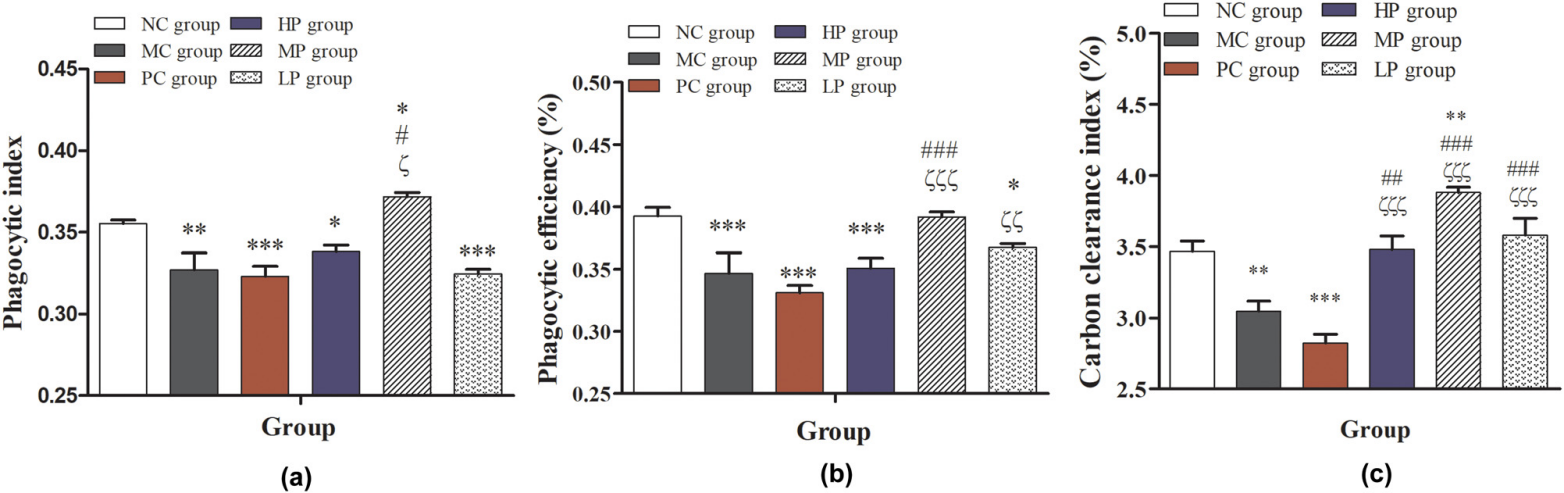
Effects of DESP on phagocytosis of macrophages in immunosuppressed mice: (a) phagocytic index, (b) phagocytic efficiency, and (c) carbon clearance index. Compared with the NC group, ^*^ *P* < 0.05, ^**^ *P* < 0.01, and ^***^ *P* < 0.001; compared with the MC group, ^#^ *P* < 0.05, ^##^ *P* < 0.01, and ^###^ *P* < 0.001; compared with the PC group, ^ζ^ *P* < 0.05, ^ζζ^ *P* < 0.01, and ^ζζζ^ *P* < 0.001.

### 3.4 Effects of DESP on function of macrophages

As shown in Figure 5a and b, the phagocytic index and phagocytic efficiency of peritoneal macrophages in MC, PC, HP, and LP groups were significantly decreased (*P* < 0.05), but the phagocytic index of peritoneal macrophages in the MP group was significantly increased (*P* < 0.05) compared with the NC group. In the DESP treatment groups, the phagocytic index and phagocytic efficiency of peritoneal macrophages were significantly higher than those in the MC and PC group (*P* < 0.001 or *P* < 0.001), only in the MP group. This indicated that middle-dose DESP could significantly increase the phagocytic activity of peritoneal macrophages in immunocompromised mice. As shown in Figure 5c, compared with the NC group, the carbon clearance ability was significantly decreased (*P* < 0.01, *P* < 0.001) in the MC and PC groups. DESP treatment can increase the carbon clearance ability of immunocompromised mice as the intermediate-dose DESP treatment significantly increased in particular (*P* < 0.01). Compared with MC and PC groups, the carbon clearance ability of immunocompromised mice was significantly improved with the three doses of DESP treatment (*P* < 0.001 or *P* < 0.001). This result indicated that DESP could significantly improve the phagocytosis of macrophages in immunocompromised mice and suggested that DESP treatment may improve the nonspecific immune ability of immunocompromised mice. Previous immunological assays showed that *D. indusiata* acid-soluble polysaccharides could improve phagocytosis of monocytes [7]. Thus, based on the present results, DESP could be regulating the auxiliary cell ability in the specific immune system.

**Figure 6:**
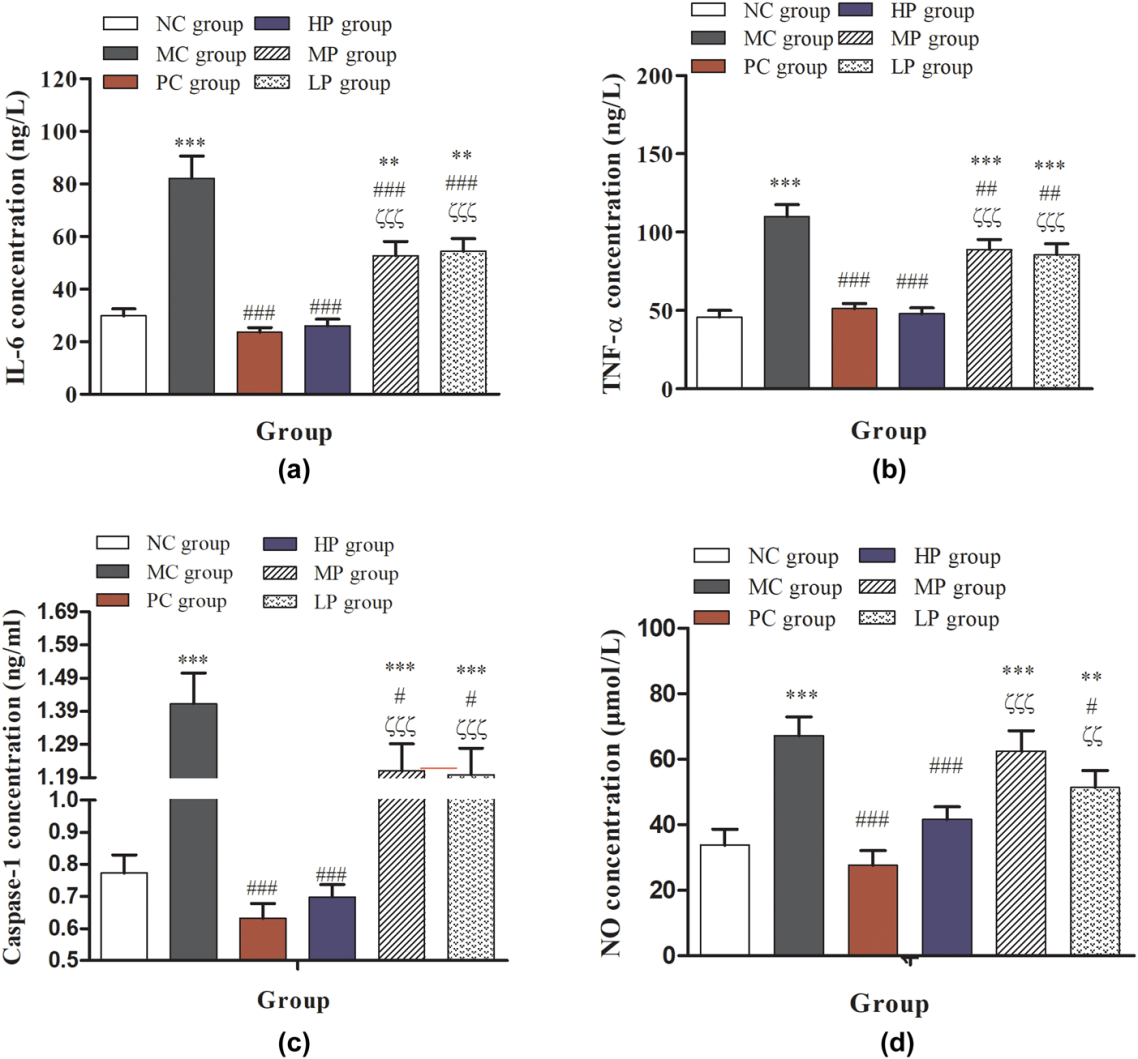
Effects of DESP on cytokine secretion of peritoneal macrophages in immunosuppressed mice: (a) IL-6 concentration, (b) TNF-α concentration, (c) caspase-1 concentration, and (d) NO concentration. Compared with the NC group, ^*^ *P* < 0.05, ^**^ *P* < 0.01, and ^***^ *P* < 0.001; compared with the MC group, ^#^ *P* < 0.05, ^##^ *P* < 0.01, and ^###^ *P* < 0.001; compared with the PC group, ^ζ^ *P* < 0.05, ^ζζ^ *P* < 0.01, and ^ζζζ^ *P* < 0.001.

As shown in Figure 6, treatment with intermediate- and low-dose DESP significantly increased the contents of IL-6, TNF-α, caspase-1, and NO secreted by peritoneal macrophages compared with NC and PC groups (*P* < 0.001). Compared with the MC group, the contents of IL-6, TNF-α, and caspase-1 secreted by peritoneal macrophages in the HP, MP, and LP groups were significantly decreased (*P* < 0.001 or *P* < 0.001). Cytokines as a signal transduction molecule between cells can regulate immune response and participate in immune cell differentiation development [39]. As shown in Figure 4f, treatment with intermediate- and low-dose DESP recovered the amount of IL-2 compared with the MC group (*P* < 0.001), rather than high-dose DESP. IL-2 as a cell growth factor promotes cell proliferation and differentiation, which can induce interferon production, which is involved in the process of autoimmune reactions [40]. TNF-α stimulates the expression of immune mediators and plays a significant role in the host defense [41]. As in previous results, the macrophages are broadly classified into M1 macrophages and M2 macrophages, which have selective anti-inflammatory, pro-fibrotic activities, and induced immunotolerance [42]. Usually, the M1 macrophages have an important ability to elevate secrete cytokines, such as IL-1β, TNF, and IL-6, and activate the inducible nitric oxide synthase generating NO [43]. Notably, the lipopolysaccharide stimulation, indeed, can induce the metabolism of arginine to NO [44]. Based on the present results, DESP had a positive regulatory effect on CTX-induced immunodeficiency in mice. The concentration of hemolysin, IL-2, TNF-α, and NO in the MP and LP groups was higher than the levels in the control group. This result shows that DESP can adjust the body’s immune imbalance and improve the symptoms of low immunity.

## 4 Discussion

The experimental results showed that different doses of DESP could change humoral immunity, cellular immunity, and nonspecific immune function of immunocompromised mice. Among them, treatment with intermediate-dose DESP significantly improved the function of macrophages. The main points are stated as follows. (1) DESP improves liver and thymus organ indexes in immunocompromised mice. (2) DESP significantly improves the delayed allergic reaction and the ability of proliferation and transformation of lymphocytes in immunocompromised mice, indicating that DESP could significantly improve the cellular immune function. (3) Intermediate-dose DESP treatment could significantly increase the content of IgA, IgG, IgM, hemolysin, IL-1β, and IL-2 in serum and enhance the humoral and cellular immune ability. (4) DESP could significantly increase the phagocytic activity and carbon clearance ability of peritoneal macrophages in immunocompromised mice, increase the speed of blood carbon clearance, and increase the contents of IL-6, TNF- α, caspase-1, and NO secreted by peritoneal macrophages, which indicated that DESP significantly improves the nonspecific immune ability of immunocompromised mice. Overall, the present results suggested that DESP can be developed as a potential health care product that can enhance immunity. However, the specific molecular mechanism of DESP treatment to improve the immune ability of immunocompromised mice needs to be further studied.

## References

[1] Ishiyama D, Fukushi Y, Ohnishi-Kameyama M, Nagata T, Mori H, Inakuma T, et al. Monoterpene-alcohols from a mushroom Dictyophora indusiata. Phytochemistry. 1999;50(6):1053–6.

[2] Lee IK, Yun BS, Han G, Cho DH, Kim YH, Yoo ID. Dictyoquinazols A, B, and C, new neuroprotective compounds from the mushroom Dictyophora indusiata. J Nat Prod. 2002;65(12):1769–72. 10.1021/np020163w 12502311

[3] Li XY, Wang ZY, Wang L, Walid E, Zhang H. In vitro antioxidant and anti-proliferation activities of polysaccharides from various extracts of different mushrooms. Int J Mol Sci. 2012;13(5):5801–17. 10.3390/ijms13055801 PMC3382807 22754332

[4] Wang J, Xu X, Zheng H, Li J, Deng C, Xu Z, et al. Structural characterization, chain conformation, and morphology of a beta-(1 → 3)-d-glucan isolated from the fruiting body of Dictyophora indusiata. J Agric Food Chem. 2009;57(13):5918–24. 10.1021/jf9009872 19530680

[5] Deng C, Fu H, Teng L, Hu Z, Xu X, Chen J, et al. Antitumor activity of the regenerated triple-helical polysaccharide from Dictyophora indusiata. Int J Biol Macromol. 2013;61:453–8. 10.1016/j.ijbiomac.2013.08.007 23973497

[6] Zhong B, Ma YS, Fu D, Zhang C. Induction of apoptosis in osteosarcoma s180 cells by polysaccharide from Dictyophora indusiata. Cell Biochem Funct. 2013;31(8):719–23. 10.1002/cbf.2961 23400947

[7] Hua Y, Gao Q, Wen L, Yang B, Tang J, You L, et al. Structural characterisation of acid- and alkali-soluble polysaccharides in the fruiting body of Dictyophora indusiata and their immunomodulatory activities. Food Chem. 2012;132(2):739–43.

[8] Wang Y, Lai L, Teng L, Li Y, Cheng J, Chen J, et al. Mechanism of the anti-inflammatory activity by a polysaccharide from Dictyophora indusiata in lipopolysaccharide-stimulated macrophages. Int J Biol Macromol. 2019;126:1158–66. 10.1016/j.ijbiomac.2019.01.022 30625352

[9] Deng C, Hu Z, Fu H, Hu M, Xu X, Chen J. Chemical analysis and antioxidant activity in vitro of a beta-d-glucan isolated from Dictyophora indusiata. Int J Biol Macromol. 2012;51(1–2):70–5. 10.1016/j.ijbiomac.2012.05.001 22579869

[10] Han S, Ma C, Hu M, Wang Y, Ma F, Tao N, et al. A polysaccharide from Dictyophora indusiata inhibits the immunosuppressive function of cancer-associated fibroblasts. Cell Biochem Funct. 2017;35(7):414–9. 10.1002/cbf.3290 28990218

[11] Hang MQ, Zou QQ, Tian HY, Sun BG, Chen HT. Analysis of volatile components from Dictyophora rubrovolota Zang, ji et liou. Procedia Eng. 2012;37:240–9.

[12] Zhuang YL, Sun LP. Nutritional characteristics of proteins from the volva and pileus in cultivated mushroom Dictyophora rubrovolvata. Int J Food Sci Nutr. 2011;62(4):392–6. 10.3109/09637486.2010.539552 21284496

[13] Wang W, Song X, Zhang J, Li H, Liu M, Gao Z, et al. Antioxidation, hepatic- and renal-protection of water-extractable polysaccharides by Dictyophora indusiata on obese mice. Int J Biol Macromol. 2019;134:290–301. 10.1016/j.ijbiomac.2019.05.028 31071398

[14] Su D, Li S, Zhang W, Wang J, Wang J, Lv M. Structural elucidation of a polysaccharide from Lonicera japonica flowers, and its neuroprotective effect on cerebral ischemia-reperfusion injury in rat. Int J Biol Macromol. 2017;99:350–7. 10.1016/j.ijbiomac.2017.02.096 28254571

[15] Zhou X, Dong Q, Kan X, Peng L, Xu X, Fang Y, et al. Immunomodulatory activity of a novel polysaccharide from Lonicera japonica in immunosuppressed mice induced by cyclophosphamide. PLoS One. 2018;13:10. 10.1371/journal.pone.0204152 PMC6175272 30296293

[16] Liang C, Tian D, Liu Y, Li H, Zhu J, Li M, et al. Review of the molecular mechanisms of Ganoderma lucidum triterpenoids: ganoderic acids A, C2, D, F, DM, X and Y. Eur J Med Chem. 2019;174:130–41. 10.1016/j.ejmech.2019.04.039 31035236

[17] Ahmad MF. Ganoderma lucidum: persuasive biologically active constituents and their health endorsement. Biomed Pharmacother. 2018;107:507–19. 10.1016/j.biopha.2018.08.036 30114634

[18] Zeng P, Guo Z, Zeng X, Hao C, Zhang Y, Zhang M, et al. Chemical, biochemical, preclinical and clinical studies of Ganoderma lucidum polysaccharide as an approved drug for treating myopathy and other diseases in China. J Cell Mol Med. 2018;22(7):3278–97. 10.1111/jcmm.13613 PMC6010762 29691994

[19] Deng C, Fu HT, Xu JJ, Shang JY, Cheng YM. Physiochemical and biological properties of phosphorylated polysaccharides from Dictyophora indusiata. Int J Biol Macromol. 2015;72:894–9. 10.1016/j.ijbiomac.2014.09.053 25316421

[20] Liao W, Lu Y, Fu J, Ning Z, Yang J, Ren J. Preparation and characterization of Dictyophora indusiata polysaccharide-zinc complex and its augmented antiproliferative activity on human cancer cells. J Agric Food Chem. 2015;63(29):6525–34. 10.1021/acs.jafc.5b00614 26155804

[21] Yu WX, Lin CQ, Zhao Q, Lin XJ, Dong XL. Neuroprotection against hydrogen peroxide-induced toxicity by Dictyophora echinovolvata polysaccharide via inhibiting the mitochondria-dependent apoptotic pathway. Biomed Pharmacother. 2017;88:569–73. 10.1016/j.biopha.2017.01.103 28135600

[22] Cohen N, Emilie A, Morisset J. Modulation of glucocorticoid-induced leucine zipper (GILZ) synthesis on macrophages functions. Immunology 2004: cytokine network, regulatory cells, signaling, and apoptosis. Malden, MA, USA: Wiley-Blackwell Publishing Ltd. 2004. p. 81–5.

[23] Berrebi D, Bruscoli S, Cohen N, Foussat A, Migliorati G, Bouchet-Delbos L, et al. Synthesis of glucocorticoid-induced leucine zipper (GILZ) by macrophages: an anti-inflammatory and immunosuppressive mechanism shared by glucocorticoids and IL-10. Blood. 2003;101(2):729–38. 10.1182/blood-2002-02-0538 12393603

[24] Li Y, Jiang W, Li ZZ, Zhang C, Huang C, Yang J, et al. Repetitive restraint stress changes spleen immune cell subsets through glucocorticoid receptor or beta-adrenergic receptor in a stage dependent manner. Biochem Biophys Res Commun. 2018;495(1):1108–14. 10.1016/j.bbrc.2017.11.148 29175389

[25] Yin YM, Fu W, Fu ML, He GQ, Traore L. The immune effects of edible fungus polysaccharides compounds in mice. Asia Pac J Clin Nutr. 2007;16:258–60. 17392115

[26] Chen XY, Zhang LN, Cheung PCK. Immunopotentiation and antitumor activity of carboxymethylated-sulfated beta-(1 → 3)-d-glucan from Poria cocos. Int Immunopharmacol. 2010;10(4):398–405. 10.1016/j.intimp.2010.01.002 20093198

[27] Ho NI, Camps MGM, de Haas EFE, Ossendorp F. Sustained cross-presentation capacity of murine splenic dendritic cell subsets in vivo. Eur J Immunol. 2018;48(7):1164–73. 10.1002/eji.201747372 PMC6055716 29676785

[28] Govindarajan S, Elewaut D, Drennan M. An Optimized Method for Isolating and Expanding Invariant Natural Killer T Cells from Mouse Spleen. J Vis Exp. 2015;105:e53256. 10.3791/53256 PMC4692670 26555769

[29] Chiba A, Cohen N, Brigl M, Brennan PJ, Besra GS, Brenner MB. Rapid and reliable generation of invariant natural killer T-cell lines in vitro. Immunology. 2009;128(3):324–33. 10.1111/j.1365-2567.2009.03130.x PMC2770680 20067532

[30] Bendelac A, Rivera MN, Park SH, Roark JH. Mouse CD1-specific NK1 T cells: development, specificity, and function. Annu Rev Immunol. 1997;15:535–62. 10.1146/annurev.immunol.15.1.535 9143699

[31] Kronenberg M, Gapin L. The unconventional lifestyle of NKT cells. Nat Rev Immunol. 2002;2(8):557–68. 10.1038/nri854 12154375

[32] Hoover SK, Barrett SK, Turk TM, Lee TC, Bear HD. Cyclophosphamide and abrogation of tumor-induced suppressor T cell activity. Cancer Immunol Immunother. 1990;31(2):121–7. 10.1007/BF01742376 PMC11038616 2138931

[33] Gathmann B, Grimbacher B, Beauté J, Dudoit Y, Mahlaoui N, Fischer A, et al. The European internet-based patient and research database for primary immunodeficiencies: results 2006–2008. Clin Exp Immunol. 2009;157:3–11. 10.1111/j.1365-2249.2009.03954.x PMC2715433 19630863

[34] Peter JG, Chapel H. Immunoglobulin replacement therapy for primary immunodeficiencies. Immunotherapy (UK). 2014;6(7):853–69. 10.2217/imt.14.54 25290417

[35] Sriaroon P, Ballow M. Immunoglobulin replacement therapy for primary immunodeficiency. Immunol Allergy Clin. 2015;35(4):713–30. 10.1016/j.iac.2015.07.006 26454315

[36] Resnick ES, Moshier EL, Godbold JH, Cunningham-Rundles C. Morbidity and mortality in common variable immune deficiency over 4 decades. Blood. 2012;119(7):1650–7. 10.1182/blood-2011-09-377945 PMC3286343 22180439

[37] Tian J, Che HL, Ha D, Wei YP, Zheng SY. Characterization and anti-allergic effect of a polysaccharide from the flower buds of Lonicera japonica. Carbohydr Polym. 2012;90(4):1642–7. 10.1016/j.carbpol.2012.07.044 22944428

[38] Zhao HP, Han ZP, Ji XM, Luo YM. Epigenetic regulation of oxidative stress in ischemic stroke. Aging Dis. 2016;7(3):295–306. 10.14336/AD.2015.1009 PMC4898926 27330844

[39] Dirchwolf M, Podhorzer A, Marino M, Shulman C, Cartier M, Zunino M, et al. Immune dysfunction in cirrhosis: Distinct cytokines phenotypes according to cirrhosis severity. Cytokine. 2016;77:14–25. 10.1016/j.cyto.2015.10.006 26517154

[40] Rajasagi NK, Rouse BT. IL-2 complex treatment amplifies CD8(+) T cell mediated immunity following herpes simplex virus-1 infection. Microbes Infect. 2016;18(12):735–46. 10.1016/j.micinf.2016.10.010 PMC5182080 27866863

[41] Ozbey G, Gorczynski R, Erin N. Stability of cytokines in supernatants of stimulated mouse immune cells. Eur Cytokine Netw. 2014;25(2):30–4. 10.1684/ecn.2014.0353 25109830

[42] Laganà AS, Salmeri FM, Ban Frangež H, Ghezzi F, Vrtačnik-Bokal E, Granese R. Evaluation of M1 and M2 macrophages in ovarian endometriomas from women affected by endometriosis at different stages of the disease. Gynecol Endocrinol. 2020;36(5):441. 10.1080/09513590.2019.1683821 31663401

[43] Chavez-Galan L, Olleros ML, Vesin D, Garcia I. Much more than M1 and M2 macrophages, there are also CD169(+) and TCR+ macrophages. Front Immunol. 2015;6:263. 10.3389/fimmu.2015.00263 PMC4443739 26074923

[44] Yeramian A, Martin L, Arpa L, Bertran J, Soler C, McLeod C, et al. Macrophages require distinct arginine catabolism and transport systems for proliferation and for activation. Eur J Immunol. 2006;36(6):1516–26. 10.1002/eji.200535694 16703566

